# Detection of anomalies in cycling behavior with convolutional neural network and deep learning

**DOI:** 10.1186/s12544-023-00583-4

**Published:** 2023-03-23

**Authors:** Shumayla Yaqoob, Salvatore Cafiso, Giacomo Morabito, Giuseppina Pappalardo

**Affiliations:** 1 Department of Electrical, Electronic, Computer and Telecommunication Engineering, University of Catania, Catania, Italy; 2 Department of Civil Engineering and Architecture, University of Catania, Catania, Italy

**Keywords:** Bicyclist safety, GPS, Convolutional neural network, Anomaly detection

## Abstract

**Background:**

Cycling has always been considered a sustainable and healthy mode of transport. With the increasing concerns of greenhouse gases and pollution, policy makers are intended to support cycling as commuter mode of transport. Moreover, during Covid-19 period, cycling was further appreciated by citizens as an individual opportunity of mobility. Unfortunately, bicyclist safety has become a challenge with growing number of bicyclists in the 21st century. When compared to the traditional road safety network screening, availability of suitable data for bicycle based crashes is more difficult. In such framework, new technologies based smart cities may require new opportunities of data collection and analysis.

**Methods:**

This research presents bicycle data requirements and treatment to get suitable information by using GPS device. Mainly, this paper proposed a deep learning-based approach “BeST-DAD” to detect anomalies and spot dangerous points on map for bicyclist to avoid a critical safety event (CSE). BeST-DAD follows Convolutional Neural Network and Autoencoder (AE) for anomaly detection. Proposed model optimization is carried out by testing different data features and BeST-DAD parameter settings, while another comparison performance is carried out between BeST-DAD and Principal Component Analysis (PCA).

**Result:**

BeST-DAD over perform than traditional PCA statistical approaches for anomaly detection by achieving 77% of the F-score. When the trained model is tested with data from different users, 100% recall is recorded for individual user’s trained models.

**Conclusion:**

The research results support the notion that proper GPS trajectory data and deep learning classification can be applied to identify anomalies in cycling behavior.

## Introduction

Cycling is a key component of any sustainable urban mobility in terms of environment and public health and as an alternative to driving a car [42]. The Netherlands is leading the ranking in Europe with 27% of trips done by bicycle with other countries (e.g. Denmark, Belgium, and Germany) already beyond the 10% threshold. Below 5% we find countries like Norway (4, 3%), Italy (3, 3%), France (2, 7%) and Luxembourg (2%) [45]. Anyway, all of them report considerable increases in bicycle usage further pushed due to the Corona crises in 2020 [26].

**Fig.1:**
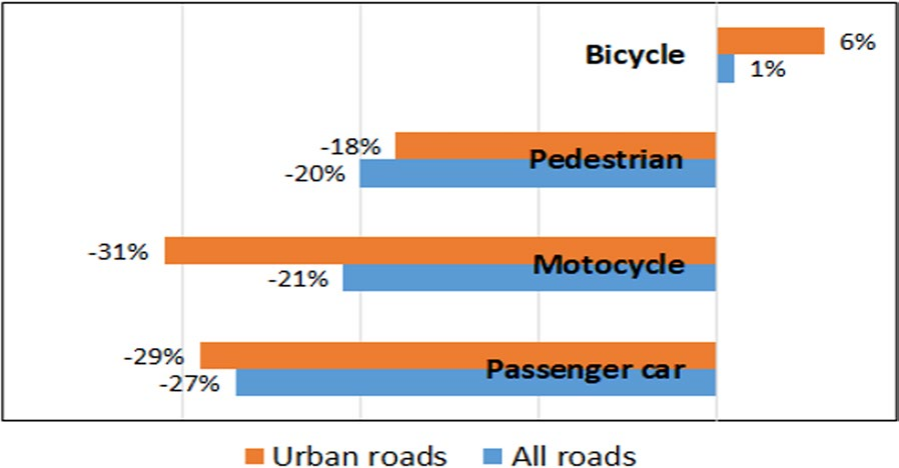
Trends 2010–2018 of fatalities in crashes involving cyclists and other transport modes. *Source*: [18]

Unfortunately, as bicycle use increases, at the same time the rate of bicycles involved in road crashes has increased, as well. Due to the vulnerability of bicyclists to serious injuries, it has been estimated that riding a bike is seven times more unsafe than travelling by car [21]. Data coming from European statistics shows that the rate of fatal accidents for cyclists in urban roads has increased from 2010 to 2018 by + 6% in contrast to the decrease of all the other modes of transport (Fig. 1) [18].

Moreover, the high risk of crashes when perceived by the users acts as strong barriers, dissuading people from using bicycles as a form of transport [46, 54]. A critical area for cycling safety research is the underreporting of cyclist crashes [29],Evelien [20] and lack of reliable data about cycling travelled distance. Even in highly cycling countries, 50% of bicycle involved traffic accidents are not reported in police statistics [22, 58]. Consequently, the crash statistics are biased in the magnitude and exposure, and less feasible than for motorized vehicles [53].

Accordingly, Traffic Conflicts, near miss or Critical Safety Events (CSEs) are spreading as crash surrogate in safety studies [2]. CSE is defined as “*A traffic event that requires a rapid evasive maneuver by the subject vehicle, or any other vehicle, pedestrian, cyclist, or animal to avoid a crash. A rapid, evasive maneuver can be steering, braking, accelerating, or combination of control inputs*” [57]. Bivariate extreme value models showed that pairs of temporal and speed-related indicators should be combined in order to properly predict severity of surrogate measures of safety [7].

Loss of control, turning, breaking and overtaking are recurrent maneuvers in CSE involving bicyclists [17, 51]. These “evasive maneuvers” are “anomalies” in the normal ride behavior whose identification is a complex task due to high dimensionality and heterogeneity trajectory data (e.g. speed, acceleration, direction) for which deep learning models for anomaly detection [61] may be more efficient than traditional statistical methods [3].

In this framework, the main contribution of this study is the development of an experimental framework where convolutional neural network (CNN) deep learning is originally applied to integrate multiple GPS data streams of bicycle kinematics to detect anomalies in the cycling behavior which are associated to evasive maneuvers in the occurrence of CSEs. Validation of the results with real data and higher performance than traditional threshold based, and statistical techniques makes the proposed approach promising in order to identify location with potential hazard for cyclists by using mobility data that can be easily collected in smart cities and communities. A case study in the city of Catania, is presented as well.

The paper is organized in the following sections:

Section 2 includes an overview of observational studies about bicyclist safety and application of CNN to road mobility and safety. Section 3 the overall method is presented in its different conceptual and operative modules which includes *Dataset preparation* (GPS data collection (typology and frequency), signal smoothing and cycling parameters calculation, data labeling into normal and abnormal) and *Neural Network Model Synthesis* (training of the convolutional autoencoder by defining architecture and setting the model parameters to perform anomaly detection). Section 4 the detection performance methodology is validated with real SCEs; different model settings are compared, and superior performance is observed over traditional detection techniques is shown. *Validation through Case study* is also carried out in Sect. 4 to demonstrate how results can be used in practical application. Section 5 is about proposed method, results and future recommendations are reported at the end of the paper.

## Related background

Section 2.1 we will provide an overview of observational studies in cyclist safety of, while in Sect. 2.2 we will focus on the research activities related to the use of deep learning and CNNs for road safety and bicyclist mobility.

### Observational studies

Literature is extensive about safety assessment using observational studies, but in comparison a limited number of studies are applied to bicyclists [2]. In the InDeV project [38], the Safe VRU app was developed for self-reporting of accidents and near-accidents and has been used by more than 400 participants [37]. The target of the UDRIVE project [49] was to identify factors in CSEs involving a bicyclist; CSEs were identified manually and correlated to the features of the infrastructure [52]. In the project BIKEALYZE, data was collected by a mobile eye tracking, a GPS-based motion data acquisition complemented with acceleration and steering direction data; CSEs (e.g. collision avoidance, way-giving violations, abrupt braking, abrupt turnout) have been identified by video-based analysis and elicitation interviews [13].

In several studies participants had an active role to indicate any CSE they experienced via a push-button on the vehicle [60] or in a smartphone app [37] or by online questionnaires [24]. A study in Sweden [16] collected movement data of 20 bicyclists with an Inertial Measurement Unit (IMU) and GPS installed on an instrumented bicycle and analyzed cycling kinematics. Longitudinal and lateral accelerations have been considered relevant for analyzing cycling behavior. Another study showed that GPS data must be collected at least with a 1 Hz frequency to provide suitable speed profiles and to detect hard breaking of cyclists [41], while vertical accelerations acquired at least at 50 Hz by accelerometer sensors are required to analyze cyclist comfort and safety due to pavement unevenness [8]. Overtaking behaviour of motorized vehicles by measuring the lateral distance between the bike and passing vehicle and a statistical model was developed to predict the probability of an unsafe critical maneuver [40] and cyclists’ safety perception [48]. In [10], authors developed an algorithm to detect a cyclist downfall by combining acceleration and rotation thresholds. Strauss & Miranda-Moreno [55] proposed a procedure to use cyclist GPS data taken by a smartphone to calculate decelerations and correlate thresholds with the number of injuries. Despite the promising results, they concluded that more granular data and validation work needs to be done to improve the reliability of the correlation. Perceived risk resulted consistently with frequency of CSEs in bicycle paths classified by a panel of experts analyzing video recording and speed and heading GPS data [9]. All these research mainly rely on the identification of safety–critical events via self-reporting, manual review of video footage pre-selected thresholds and statistical methods to analyze data.

An extensive review [5] reported that the existing solutions for trajectory outlier detection were “algorithm based” (e.g. distance-based; density-based; pattern mining–based) with emerging machine learning–based schemes that learn the outlier detection from the training trajectories to identify anomalies in the newly inserted trajectories. Moreover, the research focused more on vehicle mobility [50, 59] and not to micro-mobility, such as bicycle, which also suffer of a “digital divide” when compared to the increasing opportunities for data collection through connected and automated vehicles. More specifically, no studies are reported for cycling trajectories [5].

### Deep learning in road safety and bicyclist mobility

Recently, deep learning and Convolutional Neural Networks (CNN) have been applied in road safety studies [25, 30, 31, 39] and driving style analysis [6, 15]. The convolutional autoencoders (CAE) allowed the extraction of valuable information from large quantities of complex and heterogeneous data, showed fast convergence due to the convolutional layers, and provided better performance with multi-dimensional data compression and feature learning making the procedure well suitable for managing the mobility data characterized by high volume, variability and velocity (i.e. big Data) [36].

Dong et al. [15] made the first attempt of adopting a deep neural architecture, based on Convolutional Neural Network (CNN) and Recurrent Neural Network (RNN), to extract features on the driving style directly from GPS data. More recently, Bichicchi et al. [6] applied an unsupervised Denoising Stacked Autoencoder (SDAE) to provide output layers from kinematic measures tracked with an in-vehicle 10 Hz GPS device. The RGB colors of the outcomes were associated with different path geometries encountered during the driving.

When applied to cyclist mobility, deep learning and CNN have been used in the bike-sharing prediction modeling, because the use of shared bicycles is susceptible to time dependence and external factors [44], such as weather [4, 47], bike rebalancing and land use characteristics. In [14], authors applied the Self Organizing Map artificial neural network to identify atypical trajectories from video sequences at fixed locations. More recently in [1], authors used video records from fixed cameras and trajectory data extracted by means of computer vision algorithms and Advanced Artificial-Intelligent (AI) techniques to model cyclists’ behavior and their interactions with pedestrians in a shared space.

The limitations of the existing works for classification of abnormal cycling behavior are summarized as follows: observational studies applied on bicyclist safety mainly relay on traffic conflict techniques applied to video tracking from fixed positions. Few studies used trajectory data to identify SCE, but by self-reporting or handled classifications. In [33], authors uses text mining analytics and an Artificial Neural Network (ANN) to extract information from near-miss and collision event descriptions, acquired from BikeMaps.org, a global tool for mapping collision and near-miss events. Deep learning is becoming widely applied to transport and road safety studies, but application to cycling are mainly focused on mobility choice. Results from previous studies about CNN for anomaly detections or modeling of motorized driver behavior, cannot directly be transferred to cycling because of its specific kinematic features and limited availability of advanced equipment for data collection as in standard naturalistic studies.

To the best of our knowledge, this is the first work extending the use of deep learning CNN to extract features of the riding style of bicyclists from GPS data and to detect anomaly events in cycling behavior.

## Methodology

**Fig. 2:**
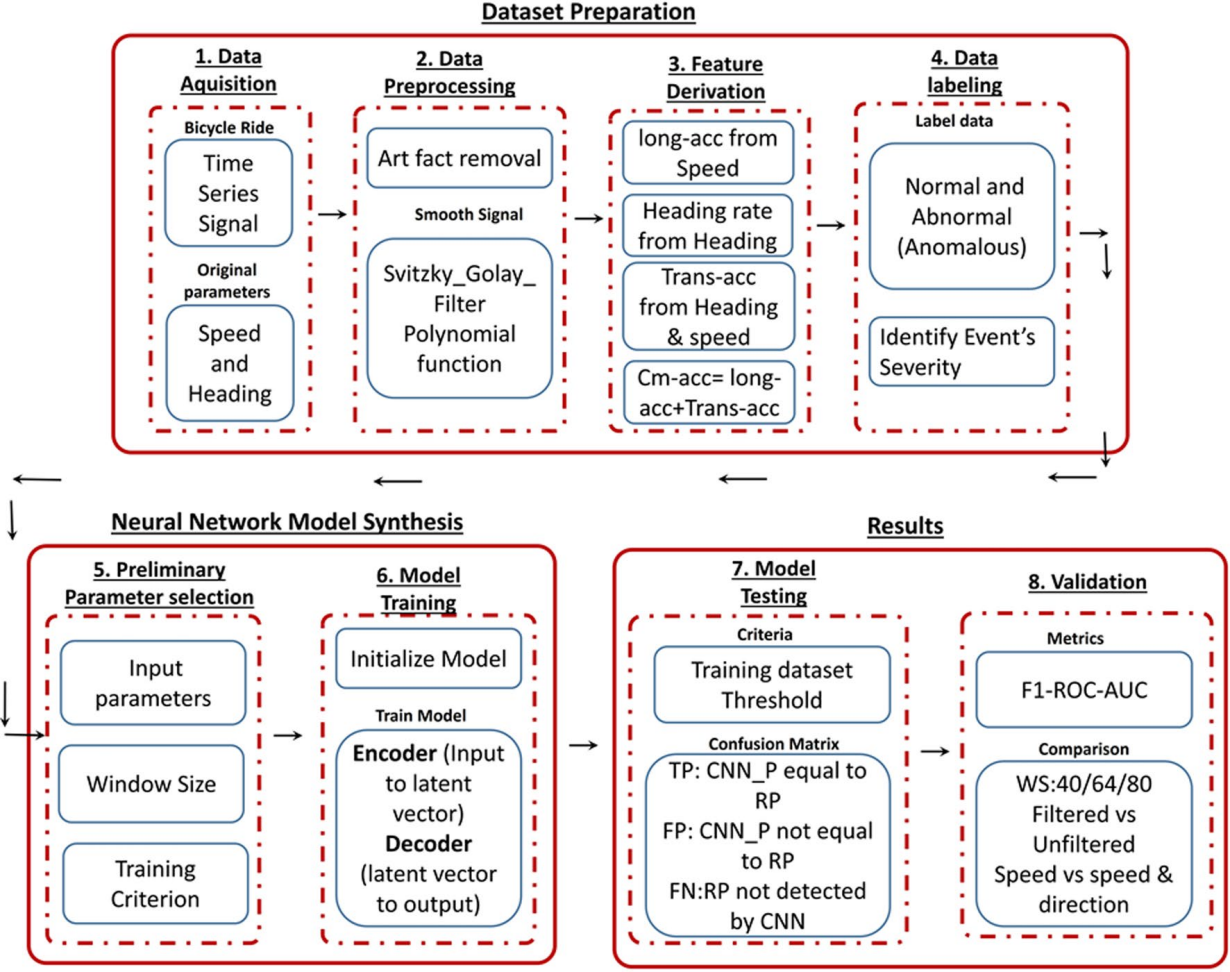
Flow diagram of method

The overall methodology consists of different tools that comprise data preparation and convolutional Neural Network training and testing as illustrated in Fig. 2.

### Dataset preparation

Dataset preparation includes both collection and treatment of GPS data to extract features optimized to train the CNN model.

#### Data acquisition

The data source is an instrumented bicycle with GPS (Global Positioning System) and HD video system (Video Vbox Lite). The Video Vbox Lite (VVL) records an extended GPS NMEA dataset not limited at only latitude, longitude, but including also speed and heading at 10 HZ frequency synchronized with a video recording of 2 HD cameras. Data accuracy and resolution are reported in Table 1. Worthily to mention that GPS in the standard acquisition without augmentation has limited position accuracy, but good data quality in speed and heading derived from the Doppler method and Carrier Phase observations [63].

**Table 1:** Accuracy and resolution of data

Data	Accuracy	Resolution
GPS Speed	0.1 km/h	0.01 km/h
GPS Heading	0.1°	0.01°
GPS 2 D position	+ 3 m 95% CEP*	(*) 95% CEP means 95% of the time the position readings will fall within a circle of the stated radius
GPS time	50 ns	1 ms
Camera	25 frame per second	720 × 576 pixels

Data was collected from 10 cyclists, named from ID-1 to ID-10, who participated in controlled test rides. The ten cyclists included eight males and two females. Participants were between 27 and 65 years of age, whereas 40% was over 40 years old. On average, the cycling experience of users was uniform with a weekly cycling use. Only ID-4 was a highly experienced commuter cyclist with daily use of bicycle. Participants were instructed to ride the instrumented bicycle following their normal behavior. The test was carried out in normal weather, daylight, and traffic hours. The ride path was long around 4 km, traveling different road infrastructures, to provide different traffic and road environment conditions that include cycle track, bicycle/bus shared line, cycle track termini and one roundabout [41]. Dataset collected for each rider contains around 9000 samples at 10 Hz acquisition frequency. Regardless of the limited number of participants such dataset is appropriate for training purposes as we will discuss in the conclusions.

**Fig. 3:**
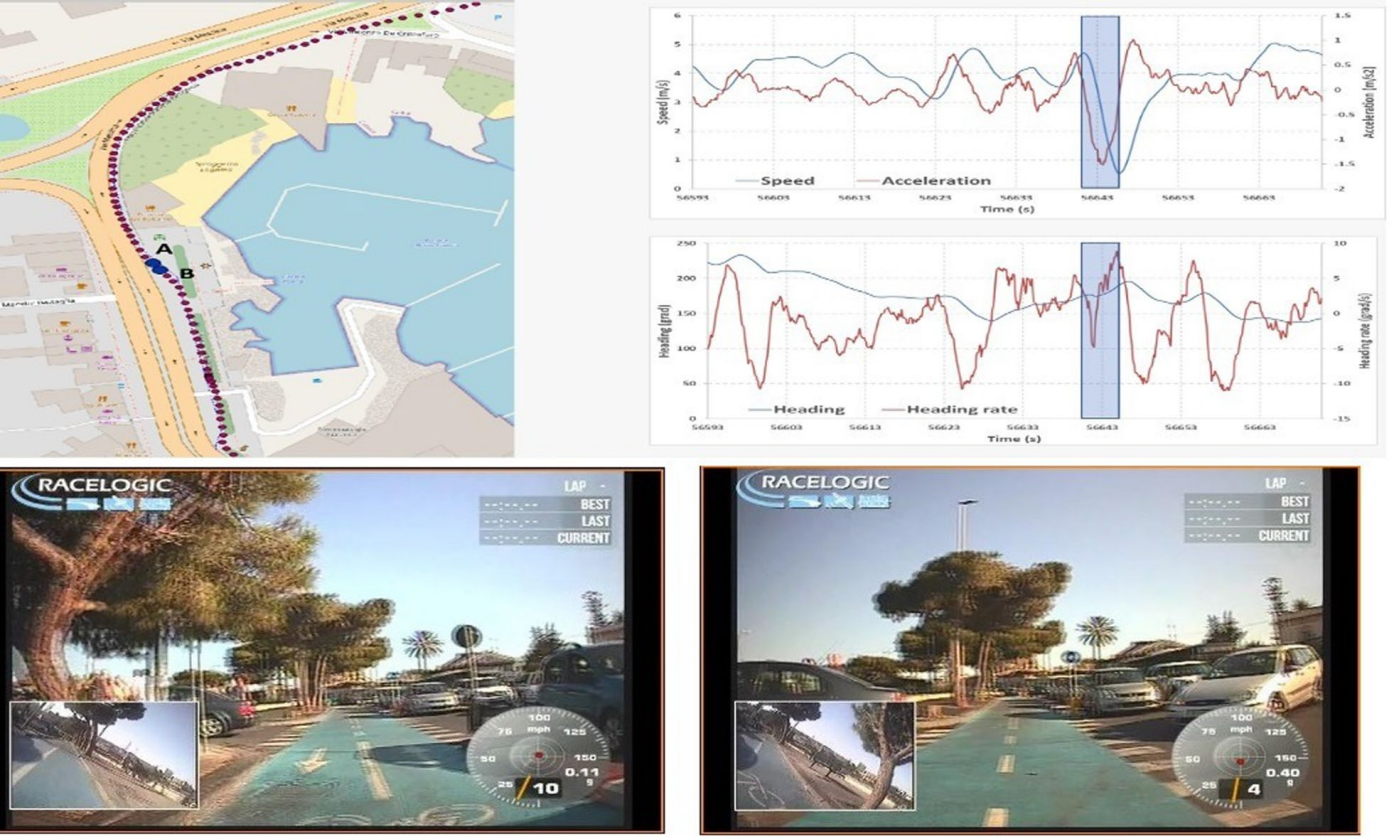
Map location along video screenshot and GPS features

CSEs occurred during the test were identified by the research team reviewing the videos together with the test rider who explained about the occurrence of an actual CSE. A total of 41 CSEs have been detected and classified over about 2.5 h of tests. As an example, Fig. 3 shows a test path section with bicyclist GPS positions and time of NMEA data. The two blue dots mark the position of one CSE, while the blue boxes show the time interval of the CSE in the speed and heading profiles. Right corner of the map includes screenshot of recorded video.

#### Data preprocessing and feature derivation

Once data was recorded, different Python routines were applied to (1) Improve the data quality, (2) Interpolate for smoothing, (3) Calculate derived parameters and (4) Create the data set for training and testing the CNN. In the present application, speed (S) and heading (H) define the recorded time data in the NMEA string, while longitudinal acceleration (LA), traveled Distance (D), heading rate (HR), transversal acceleration (TA) and combined acceleration (CA) are derived by as shown in Eqs. (1–5).

1 $$LA=\frac{\left({S}_{i+1}- {S}_{i}\right)}{\Delta T}$$

2 $$HR= \frac{\left({H}_{i+1}- {H}_{i}\right)}{\Delta T}$$

3 $$D=\frac{\left({S}_{i}+ {S}_{i+1}\right)}{2}\Delta T$$

4 $$TA = \frac{{\left[ {\left( {S_{i} + S_{i + 1} } \right)/2} \right]^{2} . HR}}{D} = \frac{{\left( {S_{i} + S_{i + 1} } \right) . \left( {H_{i + 1} - H_{i} } \right)}}{2.\Delta T}$$

5 $$CA= {\left({LA}^{2}+{TA}^{2}\right)}^{0.5}$$

where ΔT = 0.1 s, S speed in m/s, H heading in radiant, HR in rad/s, LA, LT and CA in m/s^2^.

Speed and heading data from GPS have a good standard accuracy as reported in Table 1. Anyway, environmental factors such as satellite view, signal blockage, atmospheric conditions can affect precision. Moreover, pedaling produces riding oscillation with frequencies around 2.5 Hz in the longitudinal speed and 1.2 Hz in the lateral direction [16] which can be considered as noise in the S and H signals, emphasized by the high frequency rate.

Therefore, we applied a Savitzky-Golay smoothing filter (SGF) to the speed and heading profiles, before calculating their derivatives (i.e., LA, HR, TA, CA). SGF is a digital filter [11, 32], well applied in GPS trajectory data of urban bus [34, 35], that we adapted to our time series dataset of speed and heading to increase the data precision without deforming the actual signal frequencies and shape, reducing noise and determining a smoothed trend line for deriving the other parameters.

**Fig. 4:**
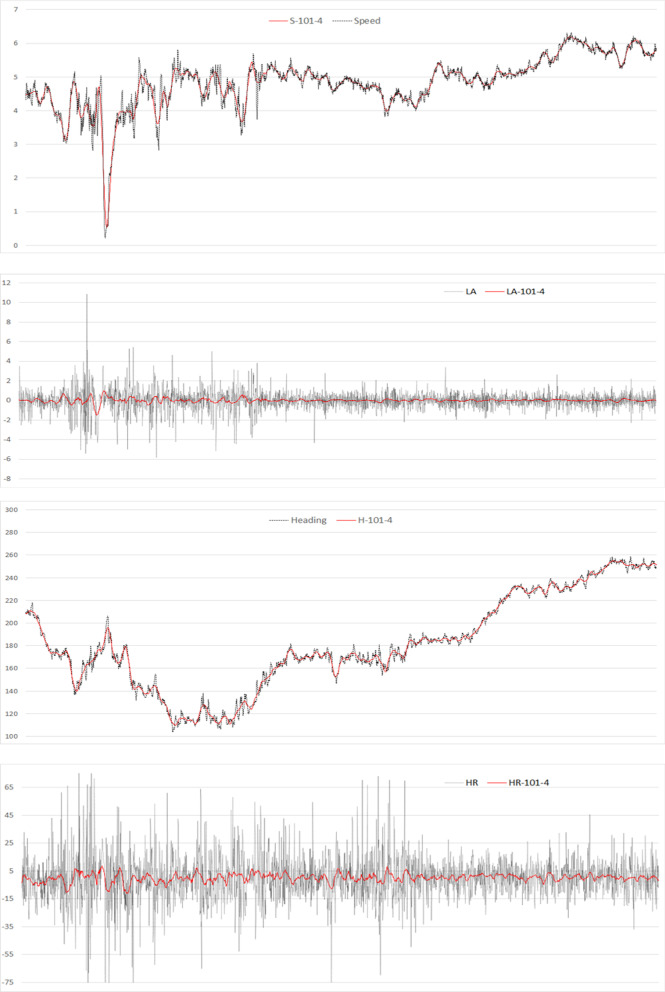
Speed, Heading with derivate LA, HR before and after SGF (101–4)

Figure 4 illustrates the original data parameters speed and direction (heading) and their derivate LA and HR before (black line) and after (red line) applying SGF. It is evident the improvement in the signal smoothness especially for the derivate of LA and HR.

### Neural network model synthesis

In this section, we propose a methodology that exploits a convolutional autoencoder for anomaly cycling detection in time series. Therefore, we will first provide CNN background and then, discussion on convolutional autoencoders (Sect. 3.2.1). Then, we will present the proposed methodology for anomaly detection in the application scenario (Sect. 3.2.2).

#### Preliminaries: convolutional neural network (CNN)

Deep learning is a recent technology used in several scenarios including the identification of anomalous points [12, 28]. Convolutional Neural Networks (CNNs) are types of deep learning algorithms, introduced to process images efficiently and are quite popular for anomaly detection as well [61, 62].

**Fig. 5:**
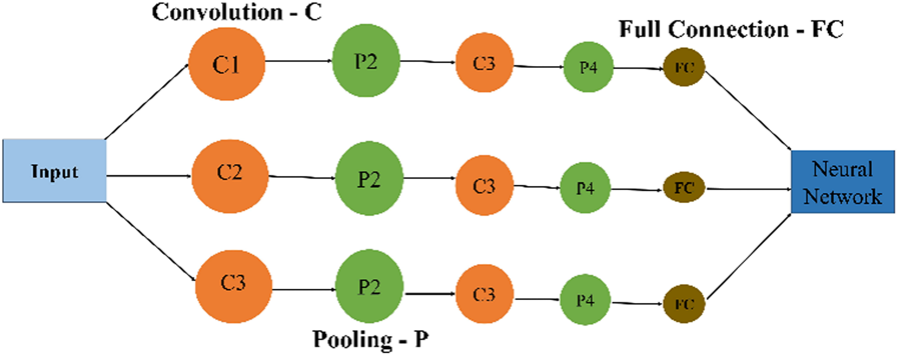
Basic CNN Architecture

CNNs automatically extract features from the data that are used for classification purposes [34, 35]. The architecture of a CNN includes several layers that are classified in convolutional layers, pooling layers, and fully connected layers (Fig. 5). The convolutional layers are the first layers of a CNN, which contains filters in the form of weighted matrix (C1) and recognize patterns efficiently by reducing the variables dimension. Convolutional layers are followed by pooling layers (S) which can be repeated several times to summarize features. The last layer is the fully connected layer whose neurons (NN) take the extracted features as their input as shown in Fig. 5.

**Fig. 6:**
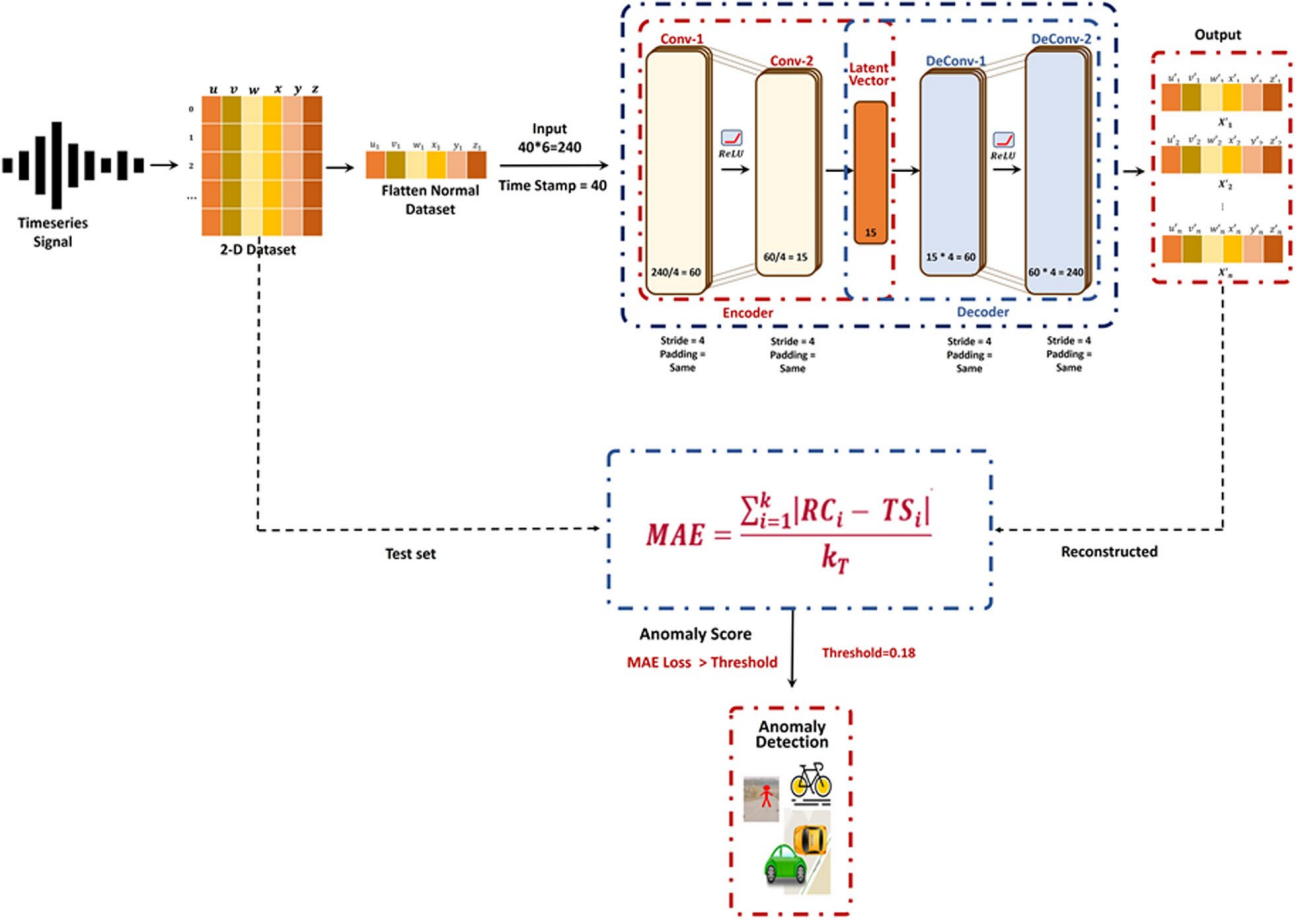
BeST-DAD scheme

In our work we use *Convolutional Autoencoders* and thus, we will provide initial information of Autoencoder (AE). An AE is a type of artificial neural network and popular for anomaly detection, AE consists of two main modules: the encoder and the decoder (Fig. 6). The encoder maps the input data into a latent vector while the decoder tries to reconstruct the input from the latent vector.

The encoder is a neural network which is specified by a set of parameters which we call ${w}_{E}$. As we already said the encoder takes as input a *n-*tuple *X* and gives as output a *m*-tuple *Z*, which we call *latent vector*, with $m\ll n$. Obviously, the latent vector *Z* is a function of the parameters ${w}_{E}$ and the input *X* as shown in Eq. (6).

6 $$Z = f_{E} \left( {w_{E} , X} \right)$$

The decoder is a neural network, specified by the parameters ${w}_{D}$ which takes as input the latent vector *Z* and gives as output a *n*-tuple $\widehat{X}$, i.e. Eq. (7). The AE training minimizes the difference between the input X and the model $\widehat{X}$ as shown in Eq. (8):

7 $$\hat{X} = f_{D} \left( {w_{D} , Z} \right) = f_{D} \left( {w_{D} ,f_{E} \left( {w_{E} ,X} \right)} \right)$$

8 $$\hat{X} \cong X \Rightarrow f_{D} \left( {w_{D} ,f_{E} \left( {w_{E} ,X} \right)} \right) \cong X$$

where $\left\| {\hat{X},X} \right\|$ represents a measure of the difference between $\hat{X}$, and X as shown in Eq. (9). Several ways of measuring such difference can be applied. Notable examples include the Mean Absolute Error (MAE), the Mean Squared Error (MSE), and the Root Mean Squared Error (RMSE). In our work we applied the MAE because it gave the best performance. This was expected; in fact, MSE and RMSE square errors before averaging, and therefore, they give higher weight to large errors. We opt MAE over Mean Square Error and Root Mean Square Error (RMSE) due to data distribution and error size suitability. Therefore,

9 $$MAE = \left\| {\hat{X},X} \right\| = \frac{1}{n}\mathop \sum \limits_{i = 1}^{n} \left| {\hat{X}_{i} - X_{i} } \right|$$

Note that convolutional autoencoders (CAE)s are capable of learning the most useful feature patterns in the input data [27] and anomaly detection [61].

#### BeST-DAD model: the proposed CNN application for anomaly detection

We call the complete scheme proposed for anomaly detection in the scenario of interest: ‘**B**icycl**e** Safety **t**hrough **D**eep learning-based **A**nomaly **D**etection’ (*BeSt-DAD)*. Best-DAD employs a 1-D CAE as depicted in (Fig. 6). The input consists of a sequence of time-data samples ${, X}_{1},{X}_{2}, \dots , {X}_{6}$ generated at a frequency of 10 Hz. The generic ${X}_{i}$ is a 6-tuple of values, i.e., ${X}_{i}=\left[{x}_{i1},{x}_{i2},{x}_{i3},{x}_{i4}, {x}_{i5},{x}_{i6}\right]$ which represent the speed, heading, heading-rate, longitudinal acceleration, transversal acceleration, combined acceleration, calculated as discussed in the previous Sect. 3.1. Thus, the input data is 2-dimensional matrix in nature, as shown in Fig. 6, that we flatten as a 1-dimensional input sequence of type as shown in Eq. (10).

10 $$x_{11} ,x_{12} ,x_{13} ,x_{14} , x_{15} ,x_{16} ,x_{21} ,x_{22} ,x_{23} ,x_{24} , x_{25} ,x_{26} , \ldots$$

The number of samples j composing the input sequence as window size. Experiments show that a good value for $j$ is $j=40$. Therefore, the input size of the encoder is $n=40\times 6=240$.

Figure 6 shows the overall architecture of BeSt-DAD where encoder consists of 2 convolutional layers. We applied Stride as an advance convolutional parameter which is capable to replace max pooling with less computation. Padding is used to maintain output dimension as input while activation function is responsible for neuron activation. In our case, each of convolutional layer reduces the input dimension of a factor equal to the stride, i.e., four. As a result, the output of the first convolutional layer has a dimension equal to 60, whereas the output of the second layer, that is, the latent vector *Z*, has dimension equal to $m=15.$

The decoder consists of two de-convolutional layers and a dropout layer which avoids model over fitting. The output of the decoder will have dimension again equal to 240 and compared to the input by calculating the mean absolute error (MAE). If this MAE is higher than a given threshold an anomaly warning is issued. Observe that the value of the such threshold is a critical parameter. We will discuss how to select it in the next section.

## Results

In the following Sect. 4, the proposed scheme will be validated by comparing the actual CSE detected as discussed in Sect. 3.1.1, which we call “real positives”, to the anomalies in cycling behavior detected by BeST-DAD, which we call “CNN positives” (Sect. 4.2). For comparison analogous validations have been carried out by using Principal Component Analysis as robust standard statistical approach for feature reduction and anomaly detection, and the more widespread method based on setting a threshold in the longitudinal acceleration to identify hard breaking. Results are presented and analysis is done in Sects. 4.3 and 4.4, based on quantitative performance metrics presented in Sect. 4.1.

### Performance metrics

In a binary classification, ‘’Positive’’ and ‘’Negative’’ assignments refer to the classifier’s prediction, and the terms ‘’True’’ and ‘’False’’ refer to whether that prediction corresponds to the real observation. Given these definitions, the confusion matrix (CM) describes the performance of the classification model as shown in Table 2.

**Table 2:** Confusion Matrix (CM)

	Real positive	Real negative
CNN positive	TP	FP
CNN negative	FN	TN

CM is useful for calculating two metrics of classification performance called *Recall* and *Precision*. Precision (P) measures the rate of true positive (TP) over the total predicted positive (TP + FP). The Recall (R) computes model’s ability to detect TP over the total number of real positives (TP + FN). For our classification with unbalanced data due to the small number of real Positive, the F-measure (F_β_) is an effective quantitative metric to select the model setting which minimizes the errors [23]. F_β_ score, in Eq. (11), is the weighted harmonic mean of precision and recall, that ranges between 0 and 1.

11 $$F_{\beta } = \frac{{\left( {1 + \beta^{2} } \right) \times P \times R}}{{\left( {\beta^{2} \times P + R} \right)}}{ }$$

As commonly used to emphasize Precision against Recall, we applied a weight β = 2, because we are more interested in limiting FN (i.e., missing detection of CSEs) rather than FP.

### Criteria for classification of CNN-positive

**Fig. 7:**
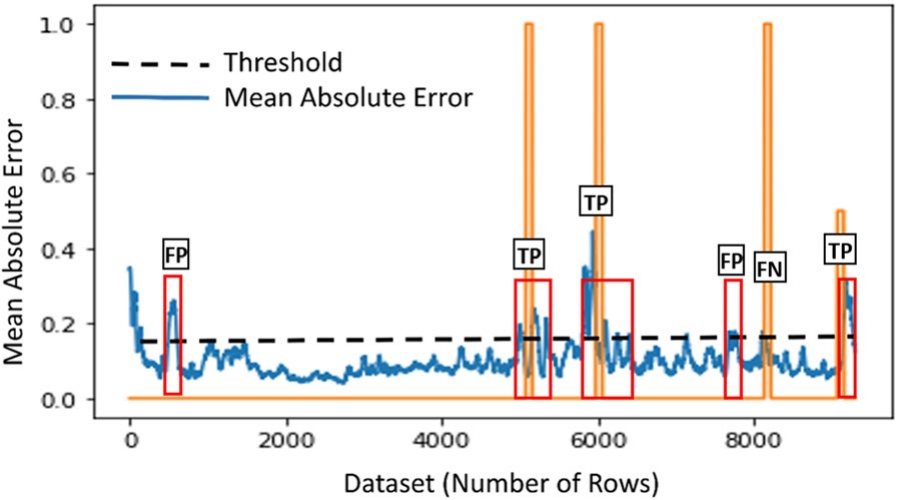
Anomaly identification example. Orange bar: Real positive; red bar: CNN positive

Our event detection criteria are illustrated in (Fig. 7) and defined as follows. Considering the observed time extension of a real CSE in the range of 0.8–3.1 s with an average of 1.4 s and the high time variability of the kinematic parameters in the cyclist riding, events with MAE > threshold of less than one second were not classified as “CNN positive” and detection sequences within 5 s have been classified as one CNN positives.

### Model testing

The model performance measured by R, P and F_2_ is affected by several factors related to the data and CNN setting and varies by changing the classification threshold. Therefore, to evaluate the results, two major comparisons are carried out: first comparison case is carried out by using various model settings and features, while other comparison case is done between BeST-DAD and alternative detection approaches like Principal Component Analysis (PCA) and breaking acceleration threshold.

To maximize the performance of the model and to learn new insights about cycling behavior modeling, model setting and features relate to the following comparisons: *CNN setting* by changing (A) Training and testing, (B) Thresholds and (C) Size of the time windows; *input dataset* by varying (D) Use of SGF filter and (E) Use of only speed related variables: speed and longitudinal acceleration or (F) Use of only heading related variables: heading and Heading rate.

Furthermore, two scenarios have been selected for training and testing of the model:

(A1) Training and Testing CNN for each user, by using 80% of data for training and 20% of data for testing. The average training time in scenario A1 is 30.142 s for each user.

(A2) Training with the data collected by considering the entire dataset related to only one user and testing with 100% of the data from all the other users. The average training time in scenario A2 is 26.642 s at all. Results and values of Recall, Precision and F_2_ are reported in Table 3.

**Table 3:** F-Score based performance evaluation for proposed scenarios

Scenario A1: Training = 80% of dataset, Testing = 20% of dataset, Preliminary Selection: Filtered data with TS_window size 40							Scenario A2: Training = 100% dataset of any one user, Testing = 100% dataset of all other users, Preliminary Selection: Filtered data with TS_window size 40						
User	T	RP	CNN	Performance metrics			User’s for training	T	RP	CNN	Performance metrics		
				Recall	Precision	F_2_					Recall	Precision	F_2_
ID-1	0.21	3	4	1	0.75	0.94	ID-1	0.18	33	47	0.76	0.53	0.70
ID-2	0.21	4	3	0.75	1	0.79	ID-2	0.18	37	39	0.70	0.67	0.69
ID-3	0.21	1	2	1	0.5	0.83	ID-3	0.18	38	47	0.76	0.62	0.73
ID-4	0.21	0	3	-	-		**ID-4**	**0.18**	**41**	**43**	**0.78**	**0.74**	**0.77**
ID-5	0.21	1	1	1	1	1.00	ID-5	0.18	38	46	0.74	0.61	0.71
ID-6	0.21	3	3	1	1	1.00	ID-6	0.18	35	47	0.77	0.57	0.72
ID-7	0.21	1	2	1	0.5	0.83	ID-7	0.18	39	49	0.80	0.65	0.76
ID-8	0.21	2	3	0.5	0.33	0.45	ID-8	0.18	34	41	0.76	0.63	0.73
ID-9	0.21	1	2	0	0	0.00	ID-9	0.18	37	45	0.73	0.60	0.70
ID-10	0.21	2	2	1	1	1.00	ID-10	0.18	37	41	0.65	0.59	0.64
**Weighted Average**	**0.21**	**18**	**26**	**0.83**	**0.60**	**0.77**	**Weighted Average**				**0.75**	**0.62**	**0.72**

The significance of bold is linked to the best result of the model

For the first scenario (A1), where each user adopts its own model, we reached the absolute best performance with an average F_2_ = 0.77. In the second scenario (A2), the model is trained with the full dataset data of one-by-one user and tested with 100% of data from the other users with an average F_2_ = 0.72. Anyway, training with ID-4 as reference user and testing with all other users gave the best F_2_ score of 0.77. While comparing the two different training approaches, we applied the best CNN settings in Table 3, with SGF, 40 TS-window, and all input parameters.

Results in Table 3 for scenario A1 show that training tailored CNN models for each user returned the best results. Anyway, in practical application, scenario A1 means the need to train BeST-DAD model for each user sharing his/her cycling data. While in scenario A2, transferring the model trained on one user returned slightly worse performances, but an overall average F_2_ comparable with the previous scenario, as well.

The previous result illustrates the reason of “ID-4” selection as 100% training dataset for the further comparisons, given the good performance and availability of a larger dataset for testing purposes (i.e., 100% of data for user ID-1, …, ID-9) by using the full set of 41 real-positives for testing.

Results for the other validation scenarios (B, C, D, E, F) are presented in Table 4. First, we tested CNN setting for different thresholds (T) and time window sizes (TW) (scenarios B, C). Results in Table 4 confirm the best performance when T = 0.18 and TW = 40. Best values of TW and T also have meaning in explaining the cycling behavior. MAE = 0.18 is equal to the 88^th^ percentile of the overall Loss values, while MAE = 0.21 is the 93^rd^ percentile, showing that anomaly cycling behaviors for evasive maneuvers are quite rare (12% of the cycling time), but not exceptional events in the riding path especially in shared lanes as will be highlighted in Sect. 5.

**Table 4:** Comparison results for different model settings and existing approach

Validation	Threshold	Performance metrics		
		Recall	Precision	F_2_
B1) Threshold = 0.15	0.15	0.80	0.54	0.73
B2) Threshold = 0.21	0.21	0.46	0.68	0.49
C1) TS_window_size = 64	0.18	0.68	0.59	0.66
C2) TS_window_size = 80	0.18	0.56	0.59	0.57
D) No SGF Filter + speed and Heading parameters	0.18	0.95	0.36	0.72
E) SGF + Only Speed parameters	0.18	0.61	0.57	0.60
F) SGF + Only Heading parameters	0.18	0.54	0.67	0.56
A1) Training each user (80/20) TS_window_size = 40, SGH + Speed parameters + Heading parameters	0.21	0.83	0.60	0.77
A2) Weighted Average of A2		0.75	0.62	0.72
PCA—training each user (80/20), TS_window_size = 40	0.17	0.66	0.34	0.45
PCA—ID-4 training (100%), TS_window_size = 40, SGH + Speed parameters + Heading parameters	0.17	0.49	0.44	0.47
Breaking Threshold—ID-4 training (100%), only Speed parameters	variable	< 0.30	< 0.30	< 0.30

Without the application of the SGF filter the high-frequency time-variability of the cycling data returned many CNN-positive with high Recall, but also many FPs returning a very low Precision (*P* = 0.36). It is worth noting that merging speed and heading parameters had the most important impact in improving the model performance. Results showed that without including heading derived parameters (i.e., HR, TA, CA), the model performance decreases significantly by missing several detections with more FNs and FPs (low R and P in Table 4). That is expected for cyclists, rather than other road users, because they apply both braking and swerving as evasive maneuver. Analyzing results, when Precision and Recall are compared it is noteworthy that Recall is always higher than Precision. This is significant because, in our application scenario missing CSE (FN) is of higher concern than False Positive. Moreover, FP may not be wrong detection of cycling anomalies, but often have been identified as changes of cycling behavior related to other events not classified as CSE (e.g., hard braking at traffic lights, avoiding pavement bumps, potholes etc.).

Finally, to further evaluate the effectiveness of deep learning in detecting CSEs, the performance metrics have been calculated also by applying a robust PCA statistic and the traditional empirical approach based on breaking acceleration threshold [56]. Results in Table 4 confirm the higher performance of deep learning for event classification.

### Validation through case study and risk assessment

**Fig. 8:**
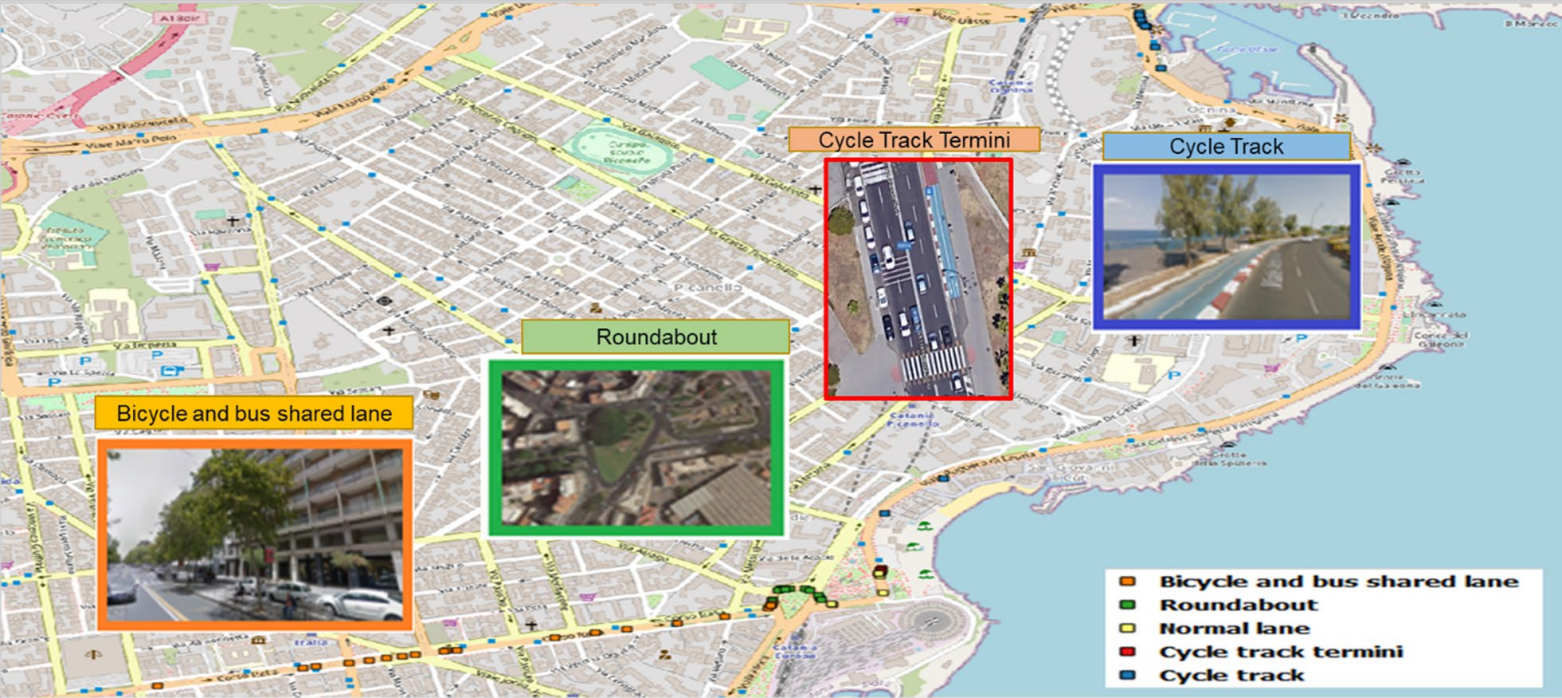
Map of BeST-DAD anomaly detections

In order to show the practical results of BeST-DAD for risk assessment and ranking, the procedure was applied to the trajectory data and anomaly detections located with the GPS coordinates in the different roadway sections composing a mixed cycling path (i.e. cycle track, roundabout, cycle track termini and bicycle/bus shared lane) (Fig. 8.).

The exposure to the occurrence of a conflict rises with the time the bicyclist spends in the road section and therefore cycling time was considered as an exposure metric to rate the number of anomalies among different roadway components to make the results comparable.

Risk Rate = N. of anomalies/cycling time.

**Fig. 9:**
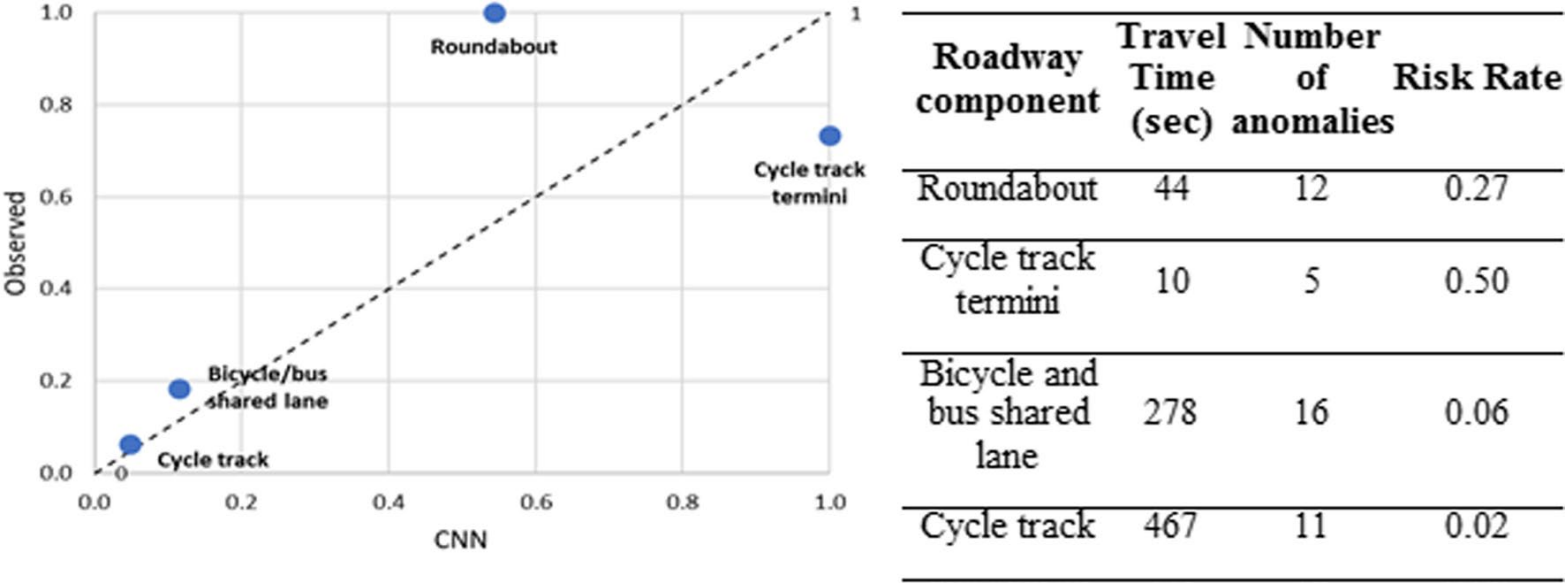
Travel Time and risk rate in various road typologies

The mean cycling time is reported in Fig. 9 which also shows the total number of BeST-DAD anomaly detections in the different road sections travelled during the test and the comparison between observed and CNN risk rates in a normalized scale (0–1).

Figure 9 shows cycle track termini at the highest risk rank followed by the roundabout. The normalized Risk Rate are also calculated to allow for comparisons with the observed risk rate reported in previous study [9]. It should be noted that in Fig. 9, the very good consistency between the two-risk ratings for cycling track and shared lane. Also consistent are the higher rating for lane termini and roundabout, even if BeST-DAD has classified a higher risk at the Cycle track termini than the one observed by actual CSE. The cycle termini end with a sharp curve before the lane crossing (Fig. 9) where cyclists were required to steer and often stop showing as anomalies in riding behavior (e.g. hard braking and steering) that have been correctly detected by BeST-DAD although not specifically related to traffic conflicts and therefore classified as FPs.

## Conclusion

Cyclists are vulnerable road users, and their safety is a serious issue to be addressed with increasing number of fatalities among cyclists. Due to the lack of reliable data for crash analysis and the opportunities to collect new data in the smart cities and bicyclist communities, innovative observational studies can offer new approaches for a network-wide safety assessment of VRUs consistent with the EU directive [19].

Auto-encoders are mainly utilized for dimensionality reduction, feature extraction, image denoising, anomaly detection and image compression [6]. In the authors- knowledge that is the first attempt to use both speed and direction GPS data with customized Convolutional Autoencoder to automatically detect anomalies in cycling behavior that can be associated to critical safety events (CSE) and plotted on map as risky points.

Performance of the classification was very good considering the low rate of FN with Recall of 100% in 6 out of 9 tests after individual training of the model (Table 3). Furthermore, in scenario A2 (Sect. 5), we have seen that also a model trained using the dataset for one selected rider can be effectively transferred to the other riders with R = 0.78 and F_2_ = 0.77. This result is interesting because, in large scale applications, the use of a pre-trained model results in the reduction of communication and energy resources and more suitable to protect the user privacy.

Performance evaluation of BeST-DAD for different model settings (Table 4) demonstrates that adding direction information (heading, heading rate, transversal acceleration) to the more traditional only speed parameters (speed and longitudinal acceleration), improved remarkably the capability of the model to detect anomalies in cycling. Data filtering by using SGF played a positive role in reducing the FPs, although CNN showed good capability to handle noise and extract features from raw input data as we observed weighted average of scenario A2 of Table 3. The advantageous application of CNN was also proven by the best performance of proposed BeST-DAD over other traditional statistical techniques like PCA or heuristic threshold-based method applied to the cyclist braking rate. A case study showed the practical application and consistency of risk assessment and ranking.

### Lessons learned and future needs

Despite the good performance of the CNN trained on the reference cyclist, we can expect larger deviance increasing the number of users. Because the CNN model depends on both the user and the specific road environment, transfer learning and cooperative learning [43] can be applied in real time to the model trained and transfer its knowledge to the specific user and road environment. To model the cycling behavior, our study used extended GPS NMEA contents (i.e. Speed, Heading) at high 10 Hz acquisition frequency which are not common in present smartphone and data mobility-data providers (e.g. Strava) following mainly the targets of profiling users' destinations and flows or fitting activity which need low frequency data acquisition (e.g. positions at 0.2 Hz). Therefore, frequencies up to 1 Hz are not yet available in standard smartphones whose development is moving mainly in the direction of improving the localization accuracy while already appropriate are speed and heading accuracy. To achieve the suitable higher frequency, an alternative to be evaluated is the capability of using SGF also for sub-sampling the GNSS signals at higher frequencies than the actual sampling rate [41].

## Data Availability

Not applicable.
